# 

**Published:** 1883-12

**Authors:** 


					﻿VcMIIfc
DECEMBER, 1883.
NO. 12.
THE
JIOMIEOPATHIC pHY£lClA]3,
A MONTHLY JOURNAL OF MEDICAL SCIENCE.
“ Magna est verilas, et prcevalebit."
IE. LIE IB, IMI. ID.,
EDITOR.
CONTENTS:
(See inside of Cover.)
PHILADELPHIA:
No. Q1O9 CHESTNUT STREET.
□ST S3 W T O B K :
A. L. CHATTERTON, PUBLISHING COMPANY, PUBLISHER’S AGENTS.
LOISTDON-:
ALFRED HEATH & CO., SOL# AGENTS FOB GREAT BRITAIN,
114 Ebury Street, Eaton Square.
Yearly Subscription, $2.50, invariably in advance.
Entered at the Philadelphia Post-Office as Second-Class Mail Matter.
				

